# A decade of partnering to stop HIV in West Africa: GAIA VF prevention, education, access to care and vaccine trial site development in Bamako, Mali

**DOI:** 10.1186/1742-4690-9-S2-P112

**Published:** 2012-09-13

**Authors:** A De Groot, K Tounkara, B Aboubacar, F Siby Diallo, F Bougoudogo, O Koita, O Koita, S Dao, S Diallo, Y Traoré, L Diarra, L Diarra, G Diarra, Y Koné, R Mali, L Steven

**Affiliations:** 1 GAIA Vaccine Foundation and the University of Rhode Island, Providence, RI, USA; 2 GAIA Vaccine Foundation, Bamako, Mali; 3 Direction Régionale de la Santé, Bamako, Mali; 4 INRSP, Bamako, Mali; 5 FMPOS, Bamako, Mali; 6 FAST U. Bamako, Bamako, Mali; 7 Hôpital Toure, Bamako, Mali; 8 ASACOMSI, Sikoro, Mali

## Background

GAIA Vaccine Foundation (GAIA VF) has been working alongside the Department of Health (DRS), HIV expert clinicians, and basic scientists in Mali to prepare a site for Phase I-III HIV vaccine trials. The proposed location for the Phase I-III trials to be conducted in this collaboration is Sikoro, a multi-ethnic village with a population of over 40,000 located near Bamako, Mali.

## Methods

To date, GAIA VF, the DRS, and Malian clinicians and scientists have collaborated on: (1) HIV education in Sikoro and Bamako, (2) HIV prevention in Sikoro, (3) access to HIV treatment in Sikoro, and (4) vaccine readiness studies in Bamako and Sikoro. Regular meetings and extensive collaborations have been established among GAIA VF, regional scientists, and government officials.

## Results

An HIV transmission prevention (MTCTP) program was established in the Sikoro prenatal care clinic (Chez Rosalie) in 2005; the Hope HIV Care Center (Bloc Espoir) was completed in 2008, and GAIA VF established the first ‘primary-care-setting’ HIV treatment program in Mali in 2009. >10,000 pregnant women have been screened for HIV infection, >200 have been diagnosed with HIV; continuing access to HIV treatment and nutritional support was made available for these women and their spouses and children in Bloc Espoir. Peer educators distributed >47,000 condoms and provided peer-to-peer education to >22,000 individuals over 5 years. >300 ‘walk-in’ patients have been screened for treatment at the on-site care center, and >195 individuals are currently receiving antiretroviral therapy in this low resource setting.

## Conclusion

GAIA VF has established successful local and regional partnerships in Mali, performed vaccine readiness research, and implemented a comprehensive HIV care program in anticipation of performing HIV vaccine trials. A Phase IV HPV vaccine trial in Mali is anticipated in 2012, which will build local expertise and capacity for future HIV vaccine trials.

